# Surveillance and analysis of adverse events following immunization (AEFI) with measles, mumps, and rubella (MMR) vaccine in Zhejiang Province, China, 2020–2025

**DOI:** 10.3389/fpubh.2026.1846354

**Published:** 2026-06-17

**Authors:** Xuejiao Pan, Yaping Chen, Hanqing He, Xiaohua Qi, Lingzhi Shen, Hui Liang

**Affiliations:** Department of Immunization Program, Zhejiang Provincial Center for Disease Control and Prevention, Hangzhou, China

**Keywords:** adverse events following immunization (AEFI), China, measles, mumps, and rubella (MMR) vaccine, surveillance and evaluation, vaccine safety

## Abstract

**Background:**

The Measles, Mumps, and Rubella (MMR) vaccine is a cornerstone of global pediatric health, yet its uptake has been declining amid growing public sensitivity about vaccine safety. This study aimed to analyze the occurrence of adverse events following immunization (AEFI) following MMR administration in Zhejiang Province from January 1, 2020, to December 31, 2025, to evaluate the vaccine's post-marketing safety profile.

**Methods:**

AEFI cases reported following MMR vaccination in Zhejiang Province between 2020 and 2025 were collected via the Chinese National Adverse Events Following Immunization Information System (CNAEFIS). Descriptive epidemiological methods were employed to analyze and evaluate the reported data.

**Results:**

From 2020 to 2025, a total of 12,597 MMR-related AEFI cases were reported, yielding an overall incidence rate of 213.15 per 100,000 doses. Among these, common adverse reactions, rare adverse reactions, and coincidental events accounted for 10,113 cases (171.12/100,000), 2,421 cases (40.96/100,000), and 63 cases (1.07/100,000), respectively. Incidence rates varied significantly by region (range: 177.30–260.45/100,000). The male-to-female ratio was 1.16:1, with 76.49% of cases occurring in children ≤1 year of age. Reports peaked during the summer and were primarily associated with the first dose. Clinical manifestations were dominated by common reactions such as fever (70.75%), rash (42.81%), and irritability (20.38%). The co-administration group exhibited significantly higher rates of high fever and rash than the MMR-only group. A total of 68 severe AEFIs were reported, with an incidence rate of 1.15 per 100,000 doses. Notably, 95.49% of cases improved without clinical intervention.

**Conclusions:**

The MMR demonstrates a safe profile in Zhejiang Province. Future efforts should focus on peak reaction periods, enhancing public communication, and strengthening primary care physician training to maintain high surveillance sensitivity and improve diagnostic and emergency response capabilities.

## 1 Introduction

As a cornerstone of global pediatric health, the Measles, Mumps, and Rubella (MMR) vaccine is essential for preventing three highly contagious viral infections (1). Notably, measles continues to be a leading cause of mortality among young children globally, causing an estimated 95,000 deaths in 2024 (2). Measles is a highly contagious, severe respiratory infection capable of causing pneumonia, encephalitis (brain swelling), and permanent deafness (3). Its high transmissibility allows a single infection to trigger exponential outbreaks in unvaccinated populations (3). Beyond acute symptoms like fever and rash, measles induces “immune amnesia,” a potent suppressive state that depletes existing antibodies and leaves children vulnerable to secondary, potentially fatal infections for years after recovery (4).

Driven by global vaccination efforts, the period between 2000 and 2018 saw a transformative 88% decline in measles incidence and a 73% reduction in mortality, marking a historic era of infectious disease control (5). However, recent years have seen a troubling reversal driven by misinformation about the vaccine, intensifying vaccine hesitancy, and COVID-19-related disruptions (6, 7). Between 2019 and 2023, measles-containing vaccine (MCV1) coverage stagnated at 80% across 104 outbreak-affected nations, triggering a 225% surge in global incidence (8, 9). The resurgence continued into 2025, with nearly 15,000 cases reported across 13 countries in the Americas, including 2,242 in the United States (10, 11). Sustained transmission in Canada led to the region losing its “measles elimination” status in November 2025 (12). These outbreaks were heavily concentrated among under-vaccinated populations, with over 90% of cases involving unvaccinated individuals or those with unknown status (13). Notably, infants under 1 year of age are the most severely affected cohort due to weakened immunity and rapid antibody waning (14).

Vaccine hesitancy remains a primary driver behind these missed immunizations—a phenomenon the WHO identified as one of the top 10 threats to global health as early as 2019 (15). Addressing this challenge requires not only robust vaccination campaigns but also rigorous safety monitoring to rebuild public trust in immunization programs (16). While rigorous pre-licensure clinical trials establish baseline safety, inherent limitations in sample size and observation periods mean that rare adverse events often only emerge during large-scale, “real-world” deployment (17). Systematic and continuous post-marketing surveillance is therefore critical to identifying causal factors and implementing timely interventions to safeguard public trust (18).

This need for localized evidence is especially pertinent given recent strategic shifts in China's Expanded Program on Immunization (EPI) (19). While the national schedule transitioned to a universal two-dose MMR regimen (at 8 and 18 months) in 2020 (19), Zhejiang Province—a highly developed coastal region—proactively implemented this change as early as December 2018 (20). Such policy adjustments inevitably intensify parental concerns and focus attention on the safety profile of the updated regimen. However, comprehensive, multi-year longitudinal data on the safety of the MMR vaccine in Zhejiang following these adjustments remain scarce. Therefore, this study analyzed all reported AEFI cases in Zhejiang Province from January 1, 2020, to December 31, 2025, to evaluate the vaccine's post-marketing safety profile, address regional health security, and provide an empirical basis for enhancing public confidence in the immunization program.

## 2 Materials and methods

### 2.1 Data sources

Case data for this study were retrieved from the Chinese National Adverse Events Following Immunization Information System (CNAEFIS). Vaccine safety monitoring in China is centralized through the CNAEFIS, a national online surveillance system where AEFI cases are systematically documented and submitted by certified physicians at the clinic level (21). We collected, verified, and cleaned all reported cases associated with MMR vaccination in Zhejiang Province from January 1, 2020, to December 31, 2025. Information on the vaccine administration and doses during the same period was collected through the Zhejiang Province Comprehensive Management Information System for Vaccines and Immunization. Demographic data were sourced from the Zhejiang Provincial Bureau of Statistics. This study was approved by the Institutional Review Board of the Zhejiang Provincial Center for Disease Control and Prevention (2026-023-01; Approval date: 12 January 2026). Due to the study's observational nature and the use of a de-identified dataset, informed consent was waived by the Ethics Committee.

### 2.2 Vaccine specifications and administration

In Zhejiang Province, the MMR vaccine is administered as part of the National Immunization Program (NIP). The specific product used is a lyophilized live attenuated vaccine formulated from the attenuated strains Hu-191 (measles), S79 (mumps), and BRD II (rubella). These strains are cultured, harvested, and blended with appropriate stabilizers before undergoing freeze-drying. In accordance with the NIP schedule in Zhejiang, children receive two 0.5 mL doses: the first at 8 months and the second at 18 months. Both are administered via subcutaneous injection in the lateral deltoid of the upper arm.

### 2.3 Case inclusion and exclusion criteria

MMR vaccine is frequently co-administered with other injectable vaccines, such as the Pneumococcal Polysaccharide Vaccine (PPV), the Diphtheria, Tetanus, and Acellular Pertussis (DTaP) vaccine, or the Japanese Encephalitis Attenuated Live Vaccine (JE-L). To ensure the specificity of the analysis, we applied the following criteria regarding co-administration to differentiate causal attribution:

Exclusion of site-specific reactions: Cases involving isolated local reactions (e.g., redness, swelling, or induration) at the injection site of a co-administered vaccine, in the absence of systemic symptoms, were attributed to the non-MMR vaccine and excluded from this study. For instance, if MMR was administered in the left upper arm and DTaP in the right, a localized induration on the right arm without accompanying systemic symptoms was considered a DTaP-related reaction and excluded.

Inclusion of systemic and indeterminate reactions: Any case presenting with systemic symptoms (e.g., fever) following co-administration, where the specific causative agent could not be differentiated, was included in the analysis. In such instances, MMR was recorded as a suspected vaccine.

### 2.4 Definitions and classifications

An AEFI is defined as a reaction or event that occurs after vaccination and is suspected to be related to the vaccine (22). Cases were classified into the following six categories based on etiology: (1) Common adverse reactions: Transient physiological dysfunctions (e.g., fever, local redness, malaise) caused by the vaccine's inherent properties. (2) Rare adverse reactions: Significant damage to tissues, organs, or functions occurring despite the use of standard vaccines and proper administration. (3) Vaccine quality accidents: Injuries resulting from substandard vaccine products. (4) Vaccination accidents: Adverse outcomes caused by violations of standardized vaccination protocols or administration guidelines. (5) Coincidental events: Illnesses that were in the incubation or prodromal stage at the time of vaccination and coincidentally manifested post-immunization. (6) Psychogenic reactions: Individual or group psychological responses (e.g., anxiety or panic) triggered by the vaccination process rather than the vaccine itself (21). The first two categories—common and rare adverse reactions—are collectively termed ‘adverse reactions,' representing the subset of AEFI cases with a potential causal association to the vaccine.

AEFI cases were dichotomized into serious and non-serious events according to standardized clinical criteria (21). Serious AEFI is defined as any event that is life-threatening, results in death, requires hospitalization (or prolongs an existing stay), or leads to persistent or significant disability or incapacity. This category also encompasses congenital anomalies or birth defects suspected to be linked to maternal vaccination during pregnancy, as well as any event requiring medical intervention to prevent these outcomes. Non-serious AEFI refers to any reaction that is not life-threatening, does not require hospitalization, and does not result in the severe adverse outcomes listed above.

### 2.5 AEFI surveillance and diagnostic procedures

CNAEFIS operates as a mandated passive surveillance system. While it relies on provider-initiated reports rather than active case-finding, the National AEFI Surveillance Guidelines strictly require all authorized healthcare personnel and vaccination units to report any suspected adverse events (23). Upon identifying an AEFI within the reporting scope, authorized reporters submit cases directly through the online system of CNAEFIS (24). Alternatively, case report cards can be submitted via mail or fax to the local County (District/City) Centers for Disease Control and Prevention (CDC), which then performs the electronic data entry into the centralized national system (24). To ensure the timeliness and sensitivity of the surveillance system, specific reporting deadlines were strictly followed. Serious AEFI cases (e.g., deaths and life-threatening conditions) were mandated to be reported within 2 h of identification, while non-serious cases were reported within 48 h (23).

The management and validation of reported cases are carried out by CDCs at various administrative levels via the web-based surveillance platform. All reported cases underwent a multi-stage review process for diagnostic classification. Standard cases are reviewed and classified by CDC personnel. For cases requiring detailed investigation (based on severity or clinical complexity), CDCs at the county, municipal, or provincial levels convene an AEFI Investigation and Diagnosis Expert Committee. These multidisciplinary panels, comprising clinicians and epidemiologists, conduct rigorous assessments of causality and definitive diagnostic classifications. In instances where the initial diagnostic conclusion is contested, formal medical appraisals or re-appraisals are performed by the municipal or provincial medical associations to ensure the accuracy and impartiality of the findings.

### 2.6 Statistical analysis

Raw AEFI data were exported to Microsoft Excel for cleaning, verification, and systematic organization. Statistical analyses were performed using R software (version 4.4.1). Descriptive statistics were employed to characterize the study population, including distributions of sex, age, dose number, reporting time, clinical symptoms, and diagnostic classifications. The AEFI incidence rate (/100,000) was calculated as the number of AEFI reports divided by total administered doses × 100,000. Comparisons of incidence rates across various sample characteristics were evaluated using the χ2 test. Trends in AEFI reporting rates were analyzed via the Cochran–Armitage test, with Bonferroni correction applied to all pairwise comparisons. A two-sided *P*-value of < 0.05 was considered statistically significant.

## 3 Results

### 3.1 MMR vaccine AEFI reporting profiles

From 2020 to 2025, a total of 5,909,900 doses of the MMR vaccine were administered in Zhejiang Province, with 12,597 AEFI cases reported. The overall cumulative incidence rate was 213.15 per 100,000 doses (95% CI: 209.46–216.90). The majority of AEFI cases (61.55%, *n* = 7,753) were reported on the same day of vaccination (Day 0), with 37.34% reported between days 1 and 9 and only 1.11% appearing after 10 days (maximum latency: 35 days).

Table 1 shows an overview of the reported MMR-related AEFIs by year and category. Common adverse reactions accounted for the majority of reports (*n* = 10,113, 80.28%), with an incidence rate of 171.12 per 100,000 doses, while rare adverse reactions accounted for 40.96 per 100,000 doses (*n* = 2,421, 19.22%). For both categories, incidence rates were significantly higher following the first dose than the second: 265.54 vs. 75.38 per 100,000 doses for common reactions (χ^2^ = 3,127.70, *P* < 0.0001) and 70.07 vs. 11.45 per 100,000 doses for rare reactions (χ^2^ = 1,240.05, *P* < 0.0001). In contrast, coincidental events were infrequent, representing a rate of only 1.07 per 100,000 doses (*n* = 63, 0.50%). No vaccine quality accidents, vaccination accidents, or psychogenic reactions were reported.

**Table 1:** Classification of AEFI cases following MMR vaccination in Zhejiang Province, 2020–2025.

Year	Doses (10^4^)	Common adverse reaction		Rare adverse reaction		Coincidental event		Total	
		**No**.	**Incidence rate** ^*^ **(95% CI)**	**No**.	**Incidence rate** ^*^ **(95% CI)**	**No**.	**Incidence rate** ^*^ **(95% CI)**	**No**.	**Incidence rate** ^*^ **(95% CI)**
2020	121.37	1,527	125.81 (119.66–132.27)	658	54.21 (50.23–58.51)	19	1.57 (1.00–2.45)	2,204	181.58 (174.16–189.32)
2021	110.8	1,234	111.37 (105.33–117.76)	427	38.54 (35.05–42.37)	7	0.63 (0.31–1.30)	1,668	150.54 (143.49–157.93)
2022	94.49	1,670	176.74 (168.47–185.42)	368	38.95 (35.17–43.13)	3	0.32 (0.11–0.93)	2,041	216.01 (206.85–225.57)
2023	92.73	1,752	188.93 (180.29–197.97)	374	40.33 (36.45–44.63)	12	1.29 (0.74–2.26)	2,138	230.55 (220.99–240.52)
2024	86.84	1,985	228.58 (218.76–238.85)	297	34.20 (30.53–38.82)	16	1.84 (1.13–2.99)	2,298	264.63 (254.04–275.65)
2025	84.76	1,945	229.48 (219.52–239.90)	297	35.04 (31.28–39.26)	6	0.71 (0.32–1.54)	2,248	265.23 (254.50–276.41)
Total	590.99	10,113	171.12 (167.82–174.48)	2,421	40.96 (39.37–42.63)	63	1.07 (0.83–1.36)	12,597	213.15 (209.46–216.90)

AEFI, adverse events following immunization; CI, confidence interval; MMR, Measles, Mumps, and Rubella. ^*^Incidence rate calculated per 100,000 doses.

**Figure 1:**
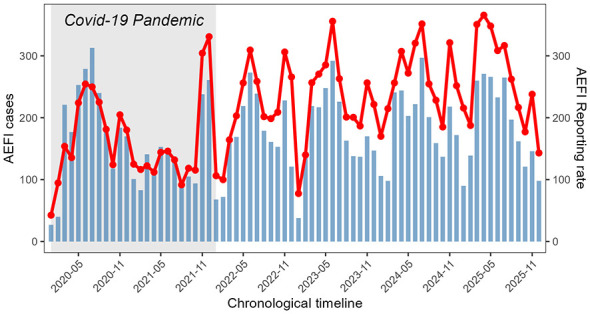
Monthly distribution of AEFI case counts and incidence rates following MMR vaccination in Zhejiang Province, 2020–2025. The blue bars represent the absolute number of reported AEFI cases (left *y*-axis), and the red line indicates the calculated AEFI reporting rate per 100,000 doses (right *y*-axis). The x-axis denotes the chronological timeline by month from January 2020 through December 2025.

While the annual number of MMR vaccine doses administered exhibited a downward trend from 2020 to 2025, the corresponding AEFI incidence rates showed a significant upward trend, rising from 181.58 per 100,000 doses in 2020 to 265.23 per 100,000 doses in 2025 (Cochran–Armitage trend test, *P* < 0.001). This increase was primarily driven by the rising reporting rate of common adverse reactions, which peaked at 229.48 per 100,000 doses in 2025. The monthly distribution of MMR-related AEFI reports from 2020 to 2025 (Figure 1) reveals a distinct seasonal pattern, with peak case counts and reporting rates consistently occurring between March and July each year. This periodicity likely aligns with annual surges in vaccination volume and “catch-up” immunization campaigns.

### 3.2 Epidemiological characteristics

The demographic analysis (Table 2) shows significant differences in AEFI incidence rates by sex, age, season, and dose sequence. Among the total cases, 6,771 were male, and 5,826 were female, with a male-to-female ratio of 1.16:1. Males had a significantly higher AEFI incidence rate than females (223.68 vs. 202.09 per 100,000 doses, *P* < 0.0001). The age distribution was predominantly in infants aged < 1 year (*n* = 9,635, 76.49%), likely associated with the established immunization schedule. The incidence rate of AEFI in the infant group was significantly higher than that of the other age groups (342.20 vs. 95.39 vs.104.47 per 100,000 doses, *P* < 0.0001). Regarding the dose sequence, the AEFI incidence rate for the first dose was significantly higher than that for the second dose (337.03 vs. 87.54 per 100,000 doses; *P* < 0.0001). Seasonally, the incidence rate ranged from 163.04 to 245.73 per 100,000 doses, with the highest rate observed in summer (*n* = 3,987), significantly higher than in the other seasons (P < 0.0001). Notably, serious AEFIs were extremely rare, accounting for only 0.54% of all reported cases, with an incidence rate of 1.15 per 100,000. doses.

**Table 2:** Demographic and clinical characteristics of AEFI cases following MMR vaccination in Zhejiang Province, 2020–2025.

Variables	AEFI cases (no.)	Percentage (%)	Incidence rate^*^(95% CI)	χ^2^	*P*-value
Sex					
Male	6,771	53.75	223.68 (218.42–229.07)	32.38	< 0.0001
Female	5,826	46.25	202.09 (196.97–207.34)		
Age (years)			
< 1	9,635	76.49	342.20 (335.45–349.09)	4,211.16	< 0.0001
1	2,842	22.56	95.39 (91.94–98.96)		
≥2	120	0.95	104.47 (87.39–124.90)		
Season			
Spring	2,246	17.83	163.04 (156.44–169.91)	248	< 0.0001
Summer	3,987	31.65	245.73 (238.23–253.47)		
Autumn	3,451	27.4	217.78 (210.64–225.16)		
Winter	2,913	23.12	219.81 (211.98–227.93)		
Dose sequence			
1st dose	10,028	79.61	337.03 (330.51–343.68)	4,323.63	< 0.0001
2nd dose	2,569	20.39	87.54 (84.22–90.99)		
Severity				—	—
Serious	68	0.54	1.15 (0.88–1.42)		
Non-serious	12,529	99.46	212.00 (208.25–215.66)		

AEFI, adverse events following immunization; CI, confidence interval; MMR, Measles, Mumps, and Rubella. ^*^Incidence rate calculated per 100,000 doses.

**Figure 2:**
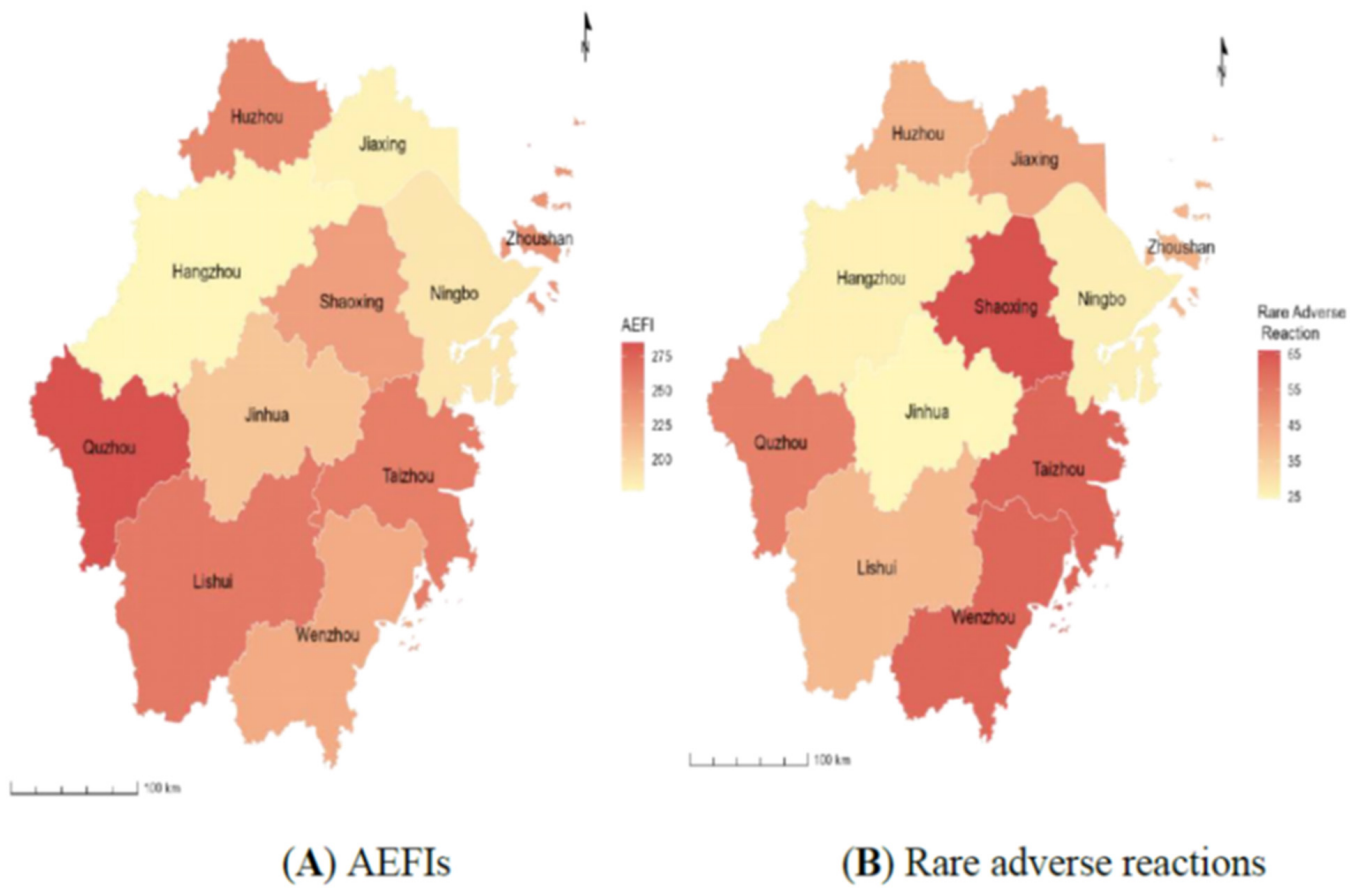
Geographic distribution of AEFI reporting rates following MMR Vaccination in Zhejiang Province, 2020–2025. The choropleth maps illustrate the regional variations in reporting rates across the 11 municipalities of Zhejiang Province. Map **(A)** displays the total AEFI incidence rates, while Map **(B)** displays the incidence rates specifically for rare adverse reactions. The color gradient represents the reporting rate per 100,000 doses, with yellow indicating the lowest rates and dark purple indicating the highest.

Regional analysis revealed geographical variation in MMR-related AEFI reporting across Zhejiang Province (Table 3, Figure 2). The highest cumulative incidence rates were concentrated in the southwestern and southeastern regions, specifically Quzhou (284.92 per 100,000 doses), followed by Lishui (260.45 per 100,000 doses) and Taizhou (257.82 per 100,000 doses). In contrast, the northern coastal regions, including Hangzhou, Jiaxing, and Ningbo, exhibited relatively lower total incidence rates. The spatial pattern for rare adverse reactions differs from the total distribution. The highest reporting rates for rare events were observed in Shaoxing (65.92 per 100,000 doses), Wenzhou (61.22 per 100,000 doses), and Taizhou (61.12 per 100,000 doses), while Jinhua recorded the lowest (24.40 per 100,000 doses). Notably, while Quzhou had the highest total AEFI rate, its rare reaction incidence rate was mid-range, suggesting that its high overall incidence may be driven primarily by common adverse reactions.

**Table 3:** Regional distribution of AEFI reporting following MMR vaccination in Zhejiang Province, 2020–2025.

Region	Total AEFI cases		Rare adverse reactions	
	**No**.	**Incidence rate** ^*^(95% CI)	**No**.	**Incidence rate** ^*^(95% CI)
Hangzhou	2,149	177.30 (169.97–184.95)	313	25.82 (23.12–28.85)
Jiaxing	897	180.48 (169.06–192.67)	227	45.67 (40.11–52.01)
Ningbo	1,584	188.80 (179.73–198.32)	222	26.46 (23.20–30.18)
Jinhua	1,695	209.92 (200.17–220.15)	197	24.40 (21.22–28.05)
Total	12,597	213.15 (209.46–216.90)	2,421	40.96 (39.37–42.63)
Wenzhou	1,928	228.32 (218.36–238.73)	517	61.22 (56.17–66.73)
Shaoxing	1,012	236.58 (222.46–251.59)	282	65.92 (58.67–74.08)
Zhoushan	165	243.12 (208.78–283.10)	28	41.26 (28.55–59.62)
Huzhou	721	251.80 (234.10–270.83)	120	41.91 (35.05–50.10)
Taizhou	1,430	257.82 (244.82–271.52)	339	61.12 (54.95–67.98)
Lishui	511	260.45 (238.85–284.00)	79	40.27 (32.31–50.17)
Quzhou	505	284.92 (261.16–310.84)	97	54.73 (44.87–66.75)

AEFI, adverse events following immunization; CI, confidence interval; MMR, Measles, Mumps, and Rubella. ^*^Incidence rate calculated per 100,000 doses.

### 3.3 Clinical characteristics

The 12,534 identified adverse reactions encompassed dozens of common clinical symptoms. Fever, rash, and irritability/crying were the three most frequently reported reactions. Specifically, 70.75% (*n* = 8,868) of cases presented with varying degrees of fever, 42.81% (*n* = 5,366) presented with a rash, and 20.38% (*n* = 2,554) presented with irritability/crying, with reporting rates of 150.05, 90.80, and 43.22 per 100,000 doses, respectively. Other observed symptoms included local reactions (redness, swelling, and induration) and systemic symptoms such as anorexia, fatigue, pruritus, and somnolence (Table 4).

**Table 4:** Clinical symptom distribution of adverse reactions following MMR vaccination in Zhejiang Province, 2020–2025.

Symptoms	Cases (no.)	Proportion (%)^a^	Incidence rate (95% CI)^b^
Fever (°C)	8,868	70.75	150.05 (146.96–153.20)
37.1–37.5	696	5.55	11.78 (10.93–12.68)
37.6–38.5	3,611	28.81	61.10 (59.14–63.13)
≥38.6	4,561	36.39	77.17 (74.97–79.45)
Rash	5,366	42.81	90.80 (88.40–93.26)
Irritability/crying	2,554	20.38	43.22 (41.57–44.92)
Redness/swelling (cm)	1,037	8.27	17.55 (16.51–18.65)
≤ 2.5	458	3.65	7.75 (7.07–8.49)
2.5–5.0	474	3.78	8.02 (7.33–8.78)
>5.0	105	0.84	1.78 (1.47–2.15)
Anorexia	650	5.19	11.00 (10.18–11.88)
Fatigue/malaise	621	4.95	10.51 (9.71–11.37)
Pruritus (Itching)	496	3.96	8.39 (7.69–9.16)
Induration (cm)	453	3.61	7.67 (6.99–8.40)
≤ 2.5	261	2.08	4.42 (3.91–4.99)
2.5–5.0	161	1.28	2.72 (2.33–3.18)
>5.0	31	0.25	0.52 (0.37–0.74)
Somnolence (drowsiness)	339	2.7	5.74 (5.16–6.38)
Abdominal pain/diarrhea	173	1.38	2.93 (2.52–3.40)
Vomiting	171	1.36	2.89 (2.49–3.36)
Hyperhidrosis (sweating)	111	0.89	1.88 (1.56–2.26)
Cough	76	0.61	1.29 (1.03–1.61)
Rhinorrhea (runny nose)	61	0.49	1.03 (0.80–1.33)
Nausea	35	0.28	0.59 (0.43–0.82)
Pharyngeal congestion	34	0.27	0.58 (0.41–0.80)
Pallor	7	0.06	0.12 (0.06–0.24)

AEFI, adverse events following immunization; CI, confidence interval. ^a^Proportion calculated as (number of specific symptoms/12,534 total adverse reactions) times 100%. ^b^Incidence rate calculated as (number of cases/5,909,900 total doses) times 100,000.

Among the 8,868 febrile cases, 51.43% (*n* = 4,561) had a body temperature ≥38.6 °C, and 28.81% (*n* = 3,611) ranged between 37.6 °C and 38.5 °C. Fever typically persisted for 1–2 days. Regarding onset, 56.92% (*n* = 5,048) occurred on the day of vaccination, followed by 11.47% (*n* = 1,017) on Day 1, and 7.17% (*n* = 636) on Day 7. While 42.56% (*n* = 3,774) presented with isolated fever, the remainder presented with one or more symptoms, such as local reactions, rash, gastrointestinal distress, or malaise.

Most cases involved the sequential appearance of rash and fever. Of the 5,366 cases involving rash, 62.56% (*n* = 3,357) occurred on the day of vaccination (including 1,530 allergic rashes, 94 urticaria, and 34 maculopapular rashes), while 6.78% (*n* = 364) appeared on Day 1 and 6.84% (*n* = 367) on Day 7. Notably, a small proportion of cases exhibited a biphasic reaction, characterized by an initial allergic rash (with or without fever) on the day of vaccination, followed by a recurrence of fever and rash between Days 6 and 10.

Since the MMR immunization schedule primarily targets infants and toddlers aged 1–2 years—a period requiring multiple scheduled vaccines—this study evaluated the impact of co-administration on AEFI profiles (Table 5). Of the 12,534 adverse reactions reported, 52.88% (*n* = 6,628) occurred following the simultaneous administration of MMR with another vaccine. While the overall incidence of general fever (≥37.1) was nearly identical between the co-administration and MMR-only groups (70.73% vs. 70.78%, *P* = 0.9557), the co-administration group exhibited significantly higher rates of high fever (≥ 38.6 °C; 38.71% vs. 33.75%, *P* < 0.0001) and rash (44.31% vs. 41.13%, *P* = 0.0003). Conversely, irritability/crying was significantly more prevalent in the MMR-only group (22.11% vs. 18.84%, *P* < 0.0001).

**Table 5:** Comparison of AEFI clinical symptoms following MMR vaccination alone vs. co-administration with other vaccines.

Clinical manifestation	MMR alone (*n* = 5,906)	Co-administration^*^(*n* = 6,628)	χ^2^	*P*-value
Fever (≥37.1 °C)	4,180 (70.78%)	4,688 (70.73%)	0.0031	0.9557
Fever (≥38.6 °C)	1,993 (33.75%)	2,566 (38.71%)	33.3226	< 0.0001
Rash	2,429 (41.13%)	2,937 (44.31%)	12.9347	0.0003
Irritability/crying	1,306 (22.11%)	1,249 (18.84%)	20.5621	< 0.0001

AEFI, adverse events following immunization; MMR, Measles, Mumps, and Rubella. ^*^Co-administration includes MMR given alongside vaccines such as Live Attenuated Japanese Encephalitis, PCV13, EV71, or Meningococcal vaccines. Percentages represent the proportion within each respective group.

### 3.4 Case outcomes and serious cases

Regarding clinical outcomes, most cases (95.49%, *n* = 12,029) improved spontaneously without medical intervention, while 4.39% (*n* = 553) achieved full recovery following treatment. Outcomes remained unknown for 13 cases due to loss of follow-up.

A total of 68 serious AEFI cases were identified during the study period, representing an overall reporting rate of 1.15 per 100,000 doses. Following investigation and diagnosis by the expert committee, 49 cases (0.83 per 100,000 doses) were classified as rare adverse reactions, accounting for 0.39% of the total AEFI reports. Of the 49 confirmed serious reactions, 33 involved co-administration with other vaccines, while 16 occurred following MMR vaccination alone. A detailed review of the 16 MMR-related serious adverse reactions (Table 6) showed that the majority (*n* = 13, 81.3%) occurred after the first dose. Immune-mediated hematological disorders, specifically Thrombocytopenic Purpura (ITP), were the most frequent serious diagnosis. Additionally, rare instances of fulminant myocarditis, febrile seizures, and laryngeal edema were recorded, most with an onset within 30 days of immunization.

**Table 6:** Serious AEFI clinical profiles (Zhejiang, 2020–2025).

Case no.	Age (years)	Sex	Vaccination date	Dose sequence	Onset date	Latency (days)	Clinical diagnosis
1	0	Male	2023/7/4	1st	2023/7/5	1	Fulminant myocarditis; Thrombocytopenia
2	0	Male	2024/4/16	1st	2024/5/6	20	ITP
3	0	Female	2024/3/27	1st	2024/3/28	1	ITP
4	0	Male	2024/9/19	1st	2024/9/19	0	Febrile seizure
5	0	Female	2023/11/28	1st	2023/12/20	22	ITP
6	0	Female	2024/11/1	1st	2024/11/25	24	ITP
7	0	Male	2024/5/18	1st	2024/5/20	2	Henoch–Schönlein purpura
8	1	Female	2025/5/19	1st	2025/5/22	3	ITP
9	0	Female	2025/6/14	1st	2025/6/21	7	ITP
10	0	Male	2025/8/19	1st	2025/8/26	7	ITP
11	0	Female	2025/9/3	1st	2025/9/10	7	Henoch–Schönlein purpura
12	1	Male	2025/8/12	1st	2025/8/12	0	Autoimmune hemolytic anemia
13	0	Female	2021/1/8	1st	2021/1/16	8	ITP
14	1	Female	2021/4/12	2nd	2021/4/12	0	Febrile seizure
15	3	Male	2022/6/16	2nd	2022/6/16	0	Laryngeal edema
16	1	Male	2022/6/11	2nd	2022/6/11	0	Febrile seizure

AEFI, adverse events following immunization; ITP, Thrombocytopenic purpura.

Two fatalities were recorded during the study period. Case 1 involved a male infant (age < 1) who received his first dose of MMR on July 4, 2023. Symptom onset occurred on July 5, and the patient died on July 31. The final clinical diagnosis was fulminant myocarditis and thrombocytopenia. Case 2 involved a female infant (age < 1) who received her first dose of MMR and her first dose of live attenuated Japanese Encephalitis (JE) vaccine on February 13, 2025. Following multiple hospitalizations beginning in March 2025, the patient died on September 27. The final clinical diagnosis included severe combined immunodeficiency (SCID), subacute sclerosing panencephalitis (SSPE), and rubella virus encephalitis.

## 4 Discussion

### 4.1 Summary of the findings

Measles remains a significant threat to global public health (2). While widespread immunization significantly reduced global mortality, the path toward total elimination is currently stalled (6, 7). In 2025–2026, several nations, including the UK and Canada, lost their “measles-eliminated” status due to declining vaccination coverage (12). This resurgence is largely driven by vaccine hesitancy, rooted in concerns over AEFI. Recent research in The Lancet (2026) (25) identifies concerns regarding side effects as a primary barrier to uptake. For hesitant populations, generic reassurances are insufficient; they require transparent, evidence-based data and expert interpretation (26). Consequently, monitoring the safety profile of the MMR vaccine is essential to maintaining public trust and sustaining high immunization coverage.

Our analysis of MMR vaccination in Zhejiang Province from 2020 to 2025 demonstrates that, although the vaccine has a high reported incidence of adverse reactions (213.15 per 100,000 doses), most of these events are mild and self-limiting. The AEFI incidence rate was significantly higher among males, infants under 1 year of age, during the summer months, and following the first dose, with significant geographical variations. The top three regions with the highest incidence rate were Quzhou, Lishui, and Taizhou. Fever and rash were the predominant symptoms, appearing in 70.75% and 42.81% of cases, respectively. The co-administration group exhibited significantly higher rates of high fever and rash than the MMR-only group. A total of 68 severe AEFIs were reported, with an incidence rate of 1.15 per 100,000 doses. Most cases (95.49%, *n* = 12,029) improved spontaneously without medical intervention.

### 4.2 Surveillance sensitivity and comparison

The incidence rate in Zhejiang Province is consistent with the annual average reported in Shanghai's Jinshan District (219.11 per 100,000) from 2011 to 2020 (27). However, it remains lower than the rate reported in Jilin Province (374.41 per 100,000) (28) and significantly higher than those in Guangzhou (60.96 per 100,000) (29) and the 2023 national average (142.58 per 100,000) (30). These discrepancies likely stem from regional variations in surveillance sensitivity across China. National data indicate that nearly half of all AEFI cases are reported from eastern provinces, with substantially lower reporting volumes in central and western regions (30). This trend is further evidenced by Zhejiang's Hib vaccine AEFI reporting rate (63.01 per 100,000) (22), which is approximately three times the national average of 22.20 per 100,000 recorded between 2021 and 2022 (31).

Additionally, in economically developed regions like Zhejiang, guardians tend to be more attentive to minor pediatric health changes, leading to more frequent clinical feedback and proactive communication with healthcare providers (22). The high public awareness and a sensitive passive monitoring system may result in higher reporting volumes. Notably, unlike previous studies that often counted only cases in which MMR was the primary “suspected vaccine,” this study included all systemic reactions in which MMR was co-administered (identified as either the first or second suspected vaccine). Because causal associations cannot be precisely distinguished during co-administration, this comprehensive inclusion strategy resulted in a reporting rate significantly higher than that in other provinces.

Consistent with global (32), national (31), and other provincial trends, AEFI reporting incidence in Zhejiang Province has shown a steady upward trend. Our findings show that the MMR vaccine reporting rate rose from 181.58 per 100,000 doses in 2020 to 265.23 per 100,000 doses by 2025. Several factors may contribute to this observed increase. First, increasing institutional emphasis on AEFI monitoring has led to improved reporting awareness and technical proficiency among healthcare providers (22). Second, frequent global vaccine-related discussions and events have intensified public scrutiny of vaccine safety (33). Third, as a passive surveillance system, the volume of reports is directly influenced by the proactive engagement of both clinicians and guardians; as clinical communication improves, the system captures a higher proportion of suspected reactions.

A substantial majority of AEFI cases (72.74%) occurred within 0–1 days of vaccination. This underscores the critical importance of immediate post-vaccination monitoring. Healthcare providers must ensure rigorous on-site observation for at least 30 minutes to manage rare but severe acute reactions, such as anaphylactic shock. Furthermore, guardians should be instructed to monitor for delayed systemic symptoms at home—specifically high fever or widespread rash—and advised to seek prompt medical attention should these acute allergic manifestations arise.

### 4.3 Epidemiological characteristics

Our analysis identified a statistically significant difference in AEFI incidence rates between males and females. While the underlying cause remains unclear, it may be attributed to greater physiological susceptibility in males or to variations in caregiver attentiveness (21). Further investigation is required to elucidate these gender-based disparities. The age distribution of reported cases was heavily concentrated in the 0-year-old group (76.49%), which aligns with the provincial immunization schedule, which includes a primary dose at 8 months. Notably, the AEFI reporting rate following the first dose (337.03 per 100,000 doses) was significantly higher than that of the second dose (87.54 per 100,000 doses), consistent with previous findings (34, 35). This trend likely reflects the robust primary immune challenge in vaccine-naive infants, the exclusion of reactive children from subsequent doses, and the natural maturation of the immune system with age (36).

The observed seasonal peak of AEFI during the summer aligns with findings from Henan (36) and Tianjin (37). This trend is likely driven by higher ambient temperatures, increased exposure of the injection site in lighter clothing, and a greater tendency for infants to scratch the area (38), all of which facilitate the detection of local reactions. The elevated AEFI reporting rates in southwestern and southeastern regions, such as Quzhou and Lishui, likely reflect enhanced rural surveillance outreach and greater diligence among local healthcare providers in these less densely populated, mountainous areas than in the northern coastal belt.

### 4.4 Clinical characteristics

In this study, common vaccine reactions accounted for 80.28% of reports, while abnormal reactions comprised 19.22%. Fever was the predominant clinical manifestation (150.05 per 100,000 doses), occurring mostly within 24 h (Days 0–1) post-vaccination, consistent with previous domestic findings (24–26). Notably, 51.43% of febrile cases involved high fever (≥38.6 °C). While most febrile episodes were self-limiting (resolving within 1–2 days), persistent high fever necessitates physical cooling or pharmacological intervention to prevent febrile seizures. Our analysis further identified a distinct biphasic reaction pattern. The acute phase (Days 0–1), often accompanied by transient rashes, likely represents a non-specific inflammatory reaction to vaccine excipients rather than viral antigens (39). Conversely, the delayed phase (Day 7) is a hallmark of “modified measles”—a benign clinical manifestation of vaccine-strain viral replication (40). Distinguishing these mechanisms is clinically vital; providing parents with this specific “anticipatory guidance” can prevent the misinterpretation of expected immunological milestones as new illnesses, reduce the risk of febrile seizures through timely intervention, and ultimately decrease unnecessary emergency department utilization.

The majority of reported rashes manifest as acute hypersensitivity reactions within 24 hours of vaccination, characterized by clinical signs of allergy such as erythema and periorbital edema. Conversely, 6.84% of rashes emerged on Day 7, presenting as sparse, mild maculopapular eruptions following the resolution of low-grade fever, occasionally accompanied by gastrointestinal symptoms (nausea/vomiting). While the former represents a reaction to vaccine excipients, the latter reflects the intended replication of the live attenuated virus. These delayed reactions align with WHO data, which estimate a 5%−10% incidence for fever and 2%−5% for transient rashes following measles-containing vaccines, typically occurring 7–10 days post-injection (41). By providing guardians with a clear chronological framework for these reactions, clinicians can effectively mitigate parental anxiety, prevent the misdiagnosis of vaccine-strain replication as a new infection, and strengthen public confidence in the immunization process.

The incidence rate of AEFI of co-administration (MMR plus a second vaccine) was significantly higher than that of MMR alone vaccination, particularly regarding high fever and rash. Notably, among the serious AEFIs, 33 out of 49 cases involved co-administration (67.34%). While the statistical spike in high fever and rash during concomitant sessions suggests a synergistic inflammatory response, it also introduces a “diagnostic shadow” that complicates causality assessment (42). In many of these cases, the adverse event may be pathophysiologically attributable to the co-administered vaccine rather than the MMR component itself (43). This implies that the standalone safety profile of the MMR vaccine is likely more favorable than the raw aggregate data suggests.

### 4.5 Serious adverse events

Serious AEFIs remained rare (0.83 per 100,000 doses), representing 0.39% of total reports. Immune-mediated conditions, specifically Thrombocytopenic Purpura (ITP) and Henoch–Schönlein Purpura, were the most common serious diagnoses. Although this rate exceeds the national average, it remains well within the WHO's projected safety parameters and is lower than rates reported in other provinces, such as Henan (3.18 per 100,000) (36). The two reported fatalities highlight the absolute necessity of pre-vaccination screening. The case involving Severe Combined Immunodeficiency (SCID) resulting in rubella encephalitis serves as a stark reminder that live attenuated vaccines are strictly contraindicated in severely immunocompromised children. Given the significant impact of SAEs on families and public trust, it is imperative to strengthen pre-vaccination screening, strictly adhere to contraindications, and maintain a cautious approach to immunization in vulnerable populations.

### 4.6 Limitations

This study is subject to several limitations that warrant consideration. First, the reliance on a passive surveillance system may not fully capture the absolute safety profile due to inherent under-reporting and variable clinical awareness. Second, because the AEFI and immunization registries are independent, the requirement for manual data entry introduces the potential for clerical errors or inconsistencies in causality assessment, despite multiple rounds of verification. Third, the lack of individual-level linkage between exposure and reaction data precluded a precisely stratified analysis of MMR alone versus co-administration. Fourth, the quality of reporting is influenced by disparate training, workloads, and “reporting hesitancy” among primary healthcare personnel. Finally, definitive evidence for causality in rare, severe events remains limited, particularly when multiple vaccines are administered simultaneously. These constraints underscore the need for future high-quality active surveillance, prospective cohort studies, or randomized clinical trials to further refine MMR safety benchmarks.

## 5 Conclusions

In conclusion, this comprehensive analysis of MMR vaccine safety in Zhejiang Province from 2020 to 2025 demonstrates a highly favorable safety profile, with the vast majority of adverse events being mild, self-limiting, and resolving without medical intervention. Serious clinical manifestations, such as ITP, remained exceedingly rare and well within established safety parameters. Given the heightened public sensitivity toward vaccine safety in recent years, it is imperative to continue strengthening AEFI monitoring and standardized management protocols. By maintaining transparent, high-quality surveillance and providing clinicians with a clear framework for managing these transient reactions, we can sustain public confidence in immunization programs and ensure the continued success of measles, mumps, and rubella elimination efforts.

## Data Availability

The raw data supporting the conclusions of this article will be made available by the authors, without undue reservation.
